# Supplementary material for: Associated Impairments among Children with Cerebral Palsy in Rural Bangladesh—Findings from the Bangladesh Cerebral Palsy Register
Source: J Clin Med. 2023 Feb 17;12(4):1597. doi: 10.3390/jcm12041597 (PMC9966186; doi:10.3390/jcm12041597)
Supplement: Supplementary file 1 [file jcm-12-01597-s001.zip › jcm-2141837-supplementary.pdf]

Supplementary files

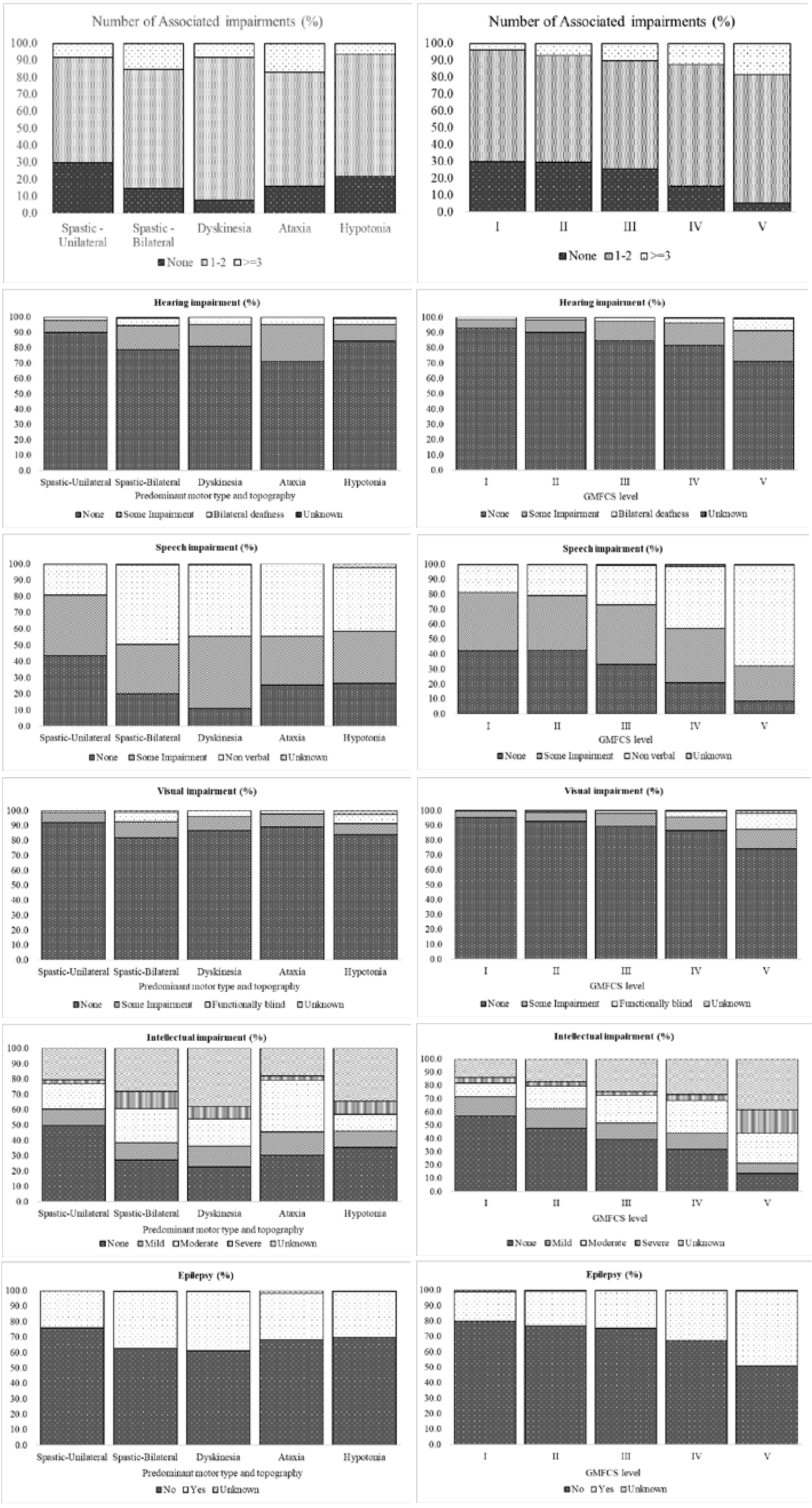

Figure S1: Presence of associated impairment among children with CP according to their predominant motor type, topography and GMFCS level

Table S1: Predictors of different types of associated impairment among children with CP in rural Bangladesh (unadjusted analysis)

Factors	Speech	Intellectual	Visual	Hearing	Epilepsy
**Antenatal care visits**					
Adequate	*Ref*	*Ref*	*Ref*	*Ref*	*Ref*
Inadequate	**0.84 (0.71-0.99)**	**0.71 (0.59-0.84)**	**0.42 (0.35-0.51)**	**0.38 (0.32-0.45)**	**0.83 (0.72-0.97)**
p value	0.04	<0.001	<0.001	<0.001	0.017
**Childbirth attended by**					
Doctor/Midwife	*Ref*	*Ref*	*Ref*	*Ref*	*Ref*
Skilled birth attendant	1.14 (0.97-1.34)	0.97 (0.82-1.15)	**0.74 (0.60-0.92)**	0.89 (0.74-1.08)	**0.66 (0.57-0.77)**
Family members	1.12 (0.90-1.41)	**1.89 (1.50-2.39)**	**1.74 (1.36-2.22)**	**2.11 (1.68-2.64)**	1.12 (0.91-1.37)
**History of birth related complications**					
No	*Ref*	*Ref*	*Ref*	*Ref*	*Ref*
Yes	**1.23 (1.07-1.43)**	**1.29 (1.10-1.50)**	1.21 (1.00-1.45)	**1.25 (1.05-1.48)**	**1.26 (1.10-1.44)**
**Gestational age (weeks)**					
Preterm (<37)	*Ref*	*Ref*	*Ref*	*Ref*	*Ref*
Term (>=37)	**1.99 (1.70-2.34)**	1.03 (0.87-1.23)	1.11 (0.89-1.38)	**1.33 (1.09-1.63)**	1.08 (0.92-1.27)
**Birthweight**					
Normal birthweight	*Ref*	*Ref*	*Ref*	*Ref*	*Ref*
Low birthweight	**0.39 (0.30-0.51)**	0.81 (0.61-1.07)	0.99 (0.70-1.39)	1.06 (0.78-1.42)	**0.76 (0.60-0.97)**
**History of febrile illness during pregnancy**					
No	*Ref*	*Ref*	*Ref*	*Ref*	*Ref*
Yes	1.06 (0.89-1.26)	0.88 (0.73-1.05)	**0.73 (0.58-0.92)**	**0.79 (0.64-0.97)**	1.08 (0.92-1.27)
**History of IPR NRD**					
No	*Ref*	*Ref*	*Ref*	*Ref*	*Ref*
Yes	**2.14 (1.83-2.51)**	**1.86 (1.56-2.22)**	**1.34 (1.08-1.68)**	1.08 (0.90-1.32)	**1.52 (1.30-1.79)**
**History of early feeding difficulties**					
No	*Ref*	*Ref*	*Ref*	*Ref*	*Ref*
Yes	**1.23 (1.07-1.43)**	**1.29 (1.10-1.50)**	1.21 (1.00-1.45)	**1.25 (1.05-1.48)**	**1.26 (1.10-1.44)**
**Timing of brain injury**					
Pre & perinatal	*Ref*	*Ref*	*Ref*	*Ref*	*Ref*
Postnatal	**1.29 (1.03-1.62)**	**3.08 (2.41-3.96)**	**2.34 (1.85-2.95)**	**3.31 (2.68-4.07)**	**1.62 (1.34-1.97)**
**Predominant motor type**					
Spastic- Unilateral	*Ref*	*Ref*	*Ref*	*Ref*	*Ref*
Spastic - Bilateral	**3.07 (2.59-3.64**	**2.72 (2.25-3.29)**	**2.38 (1.82-3.14)**	**2.35 (1.84-3.02)**	**1.85 (1.55-2.22)**
Dyskinesia	**6.15 (4.10-9.58**	**2.87 (1.97-4.22)**	1.78 (1.13-2.77)	**2.10 (1.41-3.10)**	**2.02 (1.49-2.73)**
Ataxia	**2.25 (1.59-3.25**	**2.82 (1.96-4.09)**	1.41 (0.82-2.35)	**3.65 (2.46-5.37)**	1.39 (0.97-1.98)
Hypotonia	**2.08 (1.58-2.76**	**1.42 (1.04-1.95)**	**1.86 (1.24-2.77**	**1.58 (1.08-2.29)**	**1.35 (1.01-1.79)**
**GMFCS level**					
I-II	*Ref*	*Ref*	*Ref*	*Ref*	*Ref*
III-V	**3.17 (2.72-3.70)**	**2.57 (2.17-3.03)**	**3.09 (2.39-4.05)**	**2.85 (2.28-3.60)**	**2.20 (1.87-2.59)**
**MACS level**					
I-II	*Ref*	*Ref*	*Ref*	*Ref*	*Ref*
III-V	**5.30 (4.41-6.38)**	**3.22 (2.64-3.92)**	**1.80 (1.35-2.42)**	**2.10 (1.61-2.77)**	**2.54 (2.10-3.09)**

Bold font indicates statistical significance
